# Dynamic HTA for digital health solutions: opportunities and challenges for patient-centered evaluation

**DOI:** 10.1017/S0266462323002726

**Published:** 2023-11-17

**Authors:** Jan B. Brönneke, Annika Herr, Simon Reif, Ariel D. Stern

**Affiliations:** 1 Health Innovation Hub of the German Federal Ministry of Health, Berlin, Germany; 2 Leibniz University Hannover, Hannover, Germany; 3 ZEW – Leibniz Centre for European Economic Research, Mannheim, Germany; 4 University of Erlangen-Nürnberg, Nuremberg, Germany; 5 Harvard Business School, Health Innovation Hub of the German Federal Ministry of Health, Hasso Plattner Institute, Digital Health Cluster, Harvard-MIT Center for Regulatory Science, Boston, Massachusetts, USA

**Keywords:** biomedical technology assessment, medical economics, software, government regulation, digital health, Germany, reimbursement

## Abstract

**Objectives:**

Germany’s 2019 Digital Healthcare Act (Digitale-Versorgung-Gesetz, or DVG) created a number of opportunities for the digital transformation of the healthcare delivery system. Key among these was the creation of a reimbursement pathway for patient-centered digital health applications (digitale Gesundheitsanwendungen, or DiGA). Worldwide, this is the first structured pathway for “prescribable” health applications at scale. As of October 10, 2023, 49 DiGA were listed in the official directory maintained by Germany’s Federal Institute for Drugs and Medical Devices (BfArM); these are prescribable by physicians and psychotherapists and reimbursed by the German statutory health insurance system for all its 73 million beneficiaries. Looking ahead, a major challenge facing DiGA manufacturers will be the generation of the evidence required for ongoing price negotiations and reimbursement. Current health technology assessment (HTA) methods will need to be adapted for DiGA.

**Methods:**

We describe the core issues that distinguish HTA in this setting: (i) explicit allowance for more flexible research designs, (ii) the nature of initial evidence generation, which can be delivered (in its final form) up to one year after becoming reimbursable, and (iii) the dynamic nature of both product development and product evaluation. We present the digital health applications in the German DiGA scheme as a case study and highlight the role of RWE in the successful evaluation of DiGA on an ongoing basis.

**Results:**

When a DiGA is likely to be updated and assessed regularly, full-scale RCTs are infeasible; we therefore make the case for using real-world data and real-world evidence (RWE) for *dynamic* HTAs.

**Conclusions:**

Continous evaluation using RWD is a regulatory innovation that can help improve the quality of DiGAs on the market.

## Introduction and background

### Software as a medical device

In recent years, there has been a worldwide push to articulate and clarify practices for the regulation and reimbursement of software as a medical device (SaMD). The International Medical Device Regulators Forum (IMDRF) defines SaMD as “software intended to be used for one or more medical purposes that perform these purposes without being part of a hardware medical device.” (1) The IMDRF has also begun to clarify best practices for the clinical evaluation of such products, including establishing (i) a “valid clinical association between the output of SaMD and the targeted clinical condition (to include pathological process or state)” and (ii) “that the SaMD provides the expected technical and clinical data.” (2) These two components are necessary but not sufficient conditions for the conduct of a health technology assessment (HTA). SaMD, as defined by the IMDRF, is the focus of this article; as a corollary, software embedded in a hardware medical device (which is included in some definitions, see below) will not be considered. Furthermore, SaMD not only includes native applications (“apps”) for smartphones but also all other forms of software running locally on computers or in the cloud, as well as web-based applications accessible via browsers.

### Regulatory context

The United States and Germany were two of the first countries to define details of their respective regulatory approaches to SaMD products and are the focus of this article; however, recent years have seen an expansion in the regulation of digital health applications worldwide (3). For example, England has also developed a special evidence framework for digital health technologies (4). The framework, initially based on evidence standards for “traditional” (hardware) medical devices, requires different evidence standards for different levels of potential risks and was another early mover in the regulation of SaMD products (5). In the United States, the Food and Drug Administration (FDA) has issued formal regulatory guidance related to the clinical evaluation of SaMD products (2). This guidance is based largely on the recommendations of the IMDRF and provides an outline for how such products will be evaluated by regulators. SaMD remains a key area of focus for the FDA’s digital health regulatory activities, including its Digital Health Software Precertification Program (Pre-Cert), which ran its pilot phase from 2019–2022, and the FDA’s Digital Health Center of Excellence, which was launched in 2020 (6).

Within the European Union (EU), SaMD products are regulated by the EU’s Medical Device Regulation (MDR) of 2017, which is the single, directly enforceable act of the EU, regulating the certification of all medical devices (7) for European markets; the MDR came into full effect in 2021. It covers not only hardware devices but explicitly also applies to software intended to be used for specific medical purposes (see Art. 2(1) MDR). This includes stand-alone software – SaMD – as well as software driving or influencing a medical device, regardless of its location (on a computer, a mobile phone, in the cloud, etc.) and regardless of whether it is intended to be used by health care professionals or laypersons (8). MDR follows the idea of the so-called “new approach” of the former Medical Device Directive (MDD) by principally relying on the decentralized European system of CE-certification of products that have been proven to be in conformity with harmonized standards (e.g., relevant ISO norms) and common specifications via a notified body. MDR’s requirements as well as the conformity assessment depend on the risks associated with a device. In the EU, devices are categorized into four risk classes, ranging from class I (for low-risk devices) to classes IIa and IIb (for lower and higher risk devices) to class III (for high-risk devices) (7). Requirements of the MDR cover technical and organizational issues as well as a diligent risk–benefit assessment based on a clinical evaluation of the expected medical benefit. While conformity assessments of class I devices are performed by the manufacturer, devices of classes IIa and higher require a conformity assessment and certification by an EU-designated notified body (9). With software products mostly categorized as class IIa or higher (7) in accordance with the risk assessment of the IMDRF (8), the certification requirements – including those for the clinical evaluation of a new product – are substantial. However, conformity with the MDR does not in and of itself ensure general reimbursement of new medical devices in Europe. EU member states have their own HTA processes, which govern the reimbursement of medical products and reflect their own requirements (10), making a clear and context-appropriate approach to HTA all the more important. Importantly, the HTA processes that lead to reimbursement within public healthcare systems usually have as a prerequisite that products meet MDR requirements – are only open to CE-marked medical devices – making this a necessary but not sufficient condition for reimbursement.

In the context of SaMD reimbursement, Germany, in particular, has emerged as a policy leader and innovator in recent years, with the formal establishment of policies and procedures related to digital health tools. Germany’s 2019 Digital Healthcare Act (Digitale-Versorgung-Gesetz, or DVG) created a number of opportunities for the digital transformation of the healthcare delivery system (11). Key among these was the creation of an entirely new, combined regulatory approval and reimbursement pathway for patient-centerd digital healthcare applications (in German, “digitale Gesundheitsanwendungen,” or DiGA for short) called the DiGA Fast-Track. Worldwide, this represents the first structured system for the reimbursement of “prescribable” health applications at scale.

Beyond the recent German experience, neither established frameworks for reimbursement nor HTAs of SaMD products (such as digital health applications) exist at a national level. As SaMD differs from traditional therapeutics such as pharmaceuticals and traditional (hardware-only) medical devices in several key aspects (specifically due to features such as faster research and development and continuous data processing), there is a need to move beyond the currently established, static HTA practices which assess healthcare technologies based on the existing evidence at a single point in time. This article discusses possible pathways to thoughtfully and dynamically evaluate digital health applications that are reimbursed by traditional payers (health plans). In particular, we suggest a *dynamic HTA* framework – one in which new data and evidnece can be incorporated into HTAs *as they emerge* – in a fit-for-purpose and rigorous way. We also highlight ways in which the current regulations provide tools to support its development.

## Digital health applications in Germany

### Overview

As of October 10, 2023, 49 DiGA were listed in the official Directory (“DiGA Register” or registry of approved digital health applications) maintained by Germany’s Federal Institute for Drugs and Medical Devices (BfArM). These regulated digital health applications are prescribable by physicians and psychotherapists and reimbursed by the German statutory health insurance system for all of its 73 million patients. The Digital Healthcare Application Ordinance (Digitale-Gesundheitsanwendungen-Verordnung, or DiGAV) establishes the evaluation procedures for new DiGA with respect to important criteria such as quality, data privacy, and data security, as well as requirements for establishing the so-called “positive-care effects,” a concept introduced to summarize the breadth of demonstrable benefits that such digital products can provide. The establishment of positive-care effects is crucial for listing in the BfArM Directory and therefore for both patient and reimbursement (12).

To meet the definition of a DiGA, a product must qualify as a CE-marked medical device in one of the lowest risk classes (I or IIa), primarily rely on digital technology, be intended to be directly used by patients, and be indicated for a medical purpose in accordance with the purposes stated by MDR (with the exceptions of tools for prevention, contraception, and fertility, which are circumscribed by the current legislation). Only SaMD fulfilling all criteria are eligible to apply to the DiGA directory. The novel procedure therefore bridges the gap between regulation through MDR conformity and regulation and reimbursement in the German statutory health insurance system. Importantly, the CE certification process does not include a mechanism for establishing the price of a DiGA or any other medical device. In the case of approved DiGA, prices are set by the manufacturers for the first twelve months after BfArM listing within the boundaries of a framework contract between the national associations of the manufacturers and the health insurance funds. After the first twelve months of reimbursement, a price is set by negotiation between the relevant manufacturer and the federal association of health insurance funds, or by arbitrage if an agreement cannot be reached (13).

### Prices and related considerations

As is the case for pharmaceutical products in Germany, where negotiated prices are based on a formal benefit assessment (14), the negotiated prices for digital healthcare applications are expected to deviate from initial prices and reflect their value (15). Manufacturers’ launch prices (i.e., prior to formal price negotiations) for the first 19 DiGA (i.e., those approved before July 2021) varied from €116.97 to €743.75 (mean of €415.70) per quarter. As of October 10, 2023, six DiGA had left the registry, having not provided the proposed evidence that had been a condition of their initial listing. Other DiGA manufacturers finished the first round of price negotiations after the first 12 months of reimbursement. On average, prices had increased to €453.37 for the 31 DiGA that were available one year later (June 2022). As of October 10, 2023, the average price was €406 per quarter (renewal costs are slightly lower, on average) for 48 available DiGA that are reimbursed based on a 90-day (recurring) billing cycle. One DiGA for multiple sclerosis (provisional listing since January 2023) could be bought for a singular payment of €2,077. Furthermore, the DiGA arbitration board that steps in if price negotiations fail, is currently defining the rules and price anchors for negotiations. Here, in particular, a comprehensive and appropriate HTA that is tailored to the unique facets and features of DiGA is needed for evidence-based decision-making.

While HTA procedures and best practices are well established for pharmaceutical products and traditional medical devices (albeit with recognized differences between the categories (16)), for digital health applications such practices and procedures are nascent and lack key standard features such as comparators and details on product safety and effectiveness (17). All these facts support the case for revisiting HTA in the context of DiGA to support evidence-based (and more value-based) reimbursement decisions for this growing class of healthcare products.

### A unique assessment challenge for SaMD products

While drugs are marketed in their approved form, software products have the potential to evolve – and in many cases, improve and/or expand in their scope for treatment or disease management – over time. Further, the process leading to reimbursement differs in meaningful ways. Key among them is the fact that the Fast-Track process, through which DiGA are approved by the BfArM (18), allows for applications for both permanent and preliminary listing – even before there is sufficient evidence in place to demonstrate positive-care effects. Moreover, the types of studies and evidence that are admissible in evaluating the benefit(s) of digital health applications are far broader than those that are currently used for drugs, as detailed below.

As such, articulating product-appropriate approaches to HTA in this setting is both timely and much needed. Hundreds of new digital health applications are expected to enter the German market over the coming years. As such, an appropriate and fit-for-purpose HTA will be needed both for individual products and for the entire system to deploy and use digital health applications in a value-based and evidence-driven manner.

Table 1 summarizes the 49 DiGA that became reimbursable in the German market plus the six that left the registry (as of October 10, 2023), clinical indications, launch prices, and information about the platforms through which patients can access them.

**Table 1.** Products listed in the DiGA registry as of October 10, 2023, n = 55

DiGA^a^ (+ Hardware)	Platform(s)	Indication(s)	Since	Delisted since (if yes)	Price (Gross/Quarter)	Price (rank)
Levidex^a^	Web application	Multiple sclerosis	7-Jan-23		2,077.40€ (only once)	1
Optimune^a^	Web application	Malignant neoplasm of the mammary gland	14-Jul-22		952.00 €	2
Priovi^a^	Web application	Borderline syndrome	5-Mar-23		855.82 €	3
Re.flex^a^	Apple App Store	Gonarthrosis	29-Sep-22		784.21 €	4
	Google Play Store					
sinCephalea^a^	Apple App Store	Migraine	10-Oct-22		690.00 €	5
	Google Play Store					
ProHerz^a^	Apple App Store	Heart insufficiency	15-May-23		605.00€ (first prescription)	6
	Google Play Store				495.00€ (each renewal)	
HelloBetter ratiopharm chronischer Schmerz^a^	Web application	Chronic pain	11-Dec-21		599.00 €	7
HelloBetter Schlafen^a^	Web application	Insomnia	18-Dec-22		599.00 €	
Mindable	Apple App Store	Agoraphobia and/or panic disorder	29-Apr-21		576.00 €	8
	Google Play Store					
Cara Care für Reizdarm^a^	Apple App Store	Irritable bowel syndrome	26-Dec-21		574.56 €	9
	Google Play Store					
Selfapys Online-Kurs bei Panikstörung^b^	Web application	Panic disorder	19-Jun-21	18-Nov-22	540.00 €	10
Selfapys Online-Kurs bei Binge-Eating-Störung	Apple App Store	Eating disorders	5-Jan-23		540.00 €	
	Google Play Store					
	Web application					
Selfapys Online-Kurs bei Bulimia Nervosa	Apple App Store	Bulimia	5-Jan-23		540.00 €	
	Google Play Store					
	Web application					
Selfapys Online-Kurs bei chronischen Schmerzen^a^	Apple App Store	Chronic pain	21-Apr-23		540.00 €	
	Google Play Store					
	Web application					
Elona therapy depression^a^	Apple App Store	Depression	26-Dec-22		535.49 €	11
	Google Play Store					
	Web application					
PINK! Coach^a^	Apple App Store	Malignant neoplasm of the mammary gland	27-Jun-22		533.50 €	12
	Google Play Store					
Cankado Pro-React Onco^b^	Apple App Store	Malignant neoplasm of the mammary gland [mamma]	3-May-21	21-Apr-23	499.80 €	13
	Google Play Store					
	Web application					
Vitadio^a^	Apple App Store	Diabetes mellitus, type 2	15-Apr-22		499.80 €	14
	Google Play Store					
NeuroNation^a^	Apple App Store	Cognitive disorder	13-May-23		499.00 €	15
	Google Play Store					
Mebix^a^	Apple App Store	Diabetes mellitus, type 2	14-Jul-23		499.00 €	
	Google Play Store					
Kaia Rückenschmerzen	Apple App Store	Back pain	3-Feb-23		489.39 €	16
	Google Play Store					
neolexon Aphasie^a^	Apple App Store	Aphasia and apraxia	6-Feb-22		487.90 €	17
	Google Play Store					
	Web application					
Orthopy^a^	Apple App Store	Meniscus injury	9-Sep-23		487.84 €	18
	Google Play Store					
My7steps^a^	Web application	Depression	17-Feb-23		470.05 €	19
Meine Tinnitus App – Das digitale Tinnitus Counseling^a^	Apple App Store	Tinnitus	6-Mar-22		449.00 €	20
	Google Play Store					
Rehappy^b^ (+ Optional Armband)	Apple App Store	Stroke	29-Dec-20	26-Sep-22	449.00€ (with armband)	
	Google Play Store				299.00€ (without)	
Mika^b^	Apple App Store	Malignant neoplasm of the cervix uteri, uterus, and ovary	25-Mar-21	25-Mar-22	419.00 €	21
	Google Play Store					
Kaia COPD^a^	Apple App Store	COPD	26-Dec-22		415.00 €	22
	Google Play Store					
Oviva Direkt für Adipositas	Apple App Store	Obesity	03.10.2021		411.30 €	23
	Google Play Store					
Endo-App^a^	Apple App Store	Endometriosis	9-Oct-22		402.30 €	24
	Google Play Store					
Smoke Free – Rauchen aufhören^a^	Apple App Store	Mental and behavioral disorders caused by tobacco: addiction syndrome	29-Jan-23		389.00 €	25
	Google Play Store					
Edupression^a^	Web application	Depression	26-Dec-22		357.00€ (first prescription)	26
					178.50€ (each renewal)	
companion patella powered by medi-proved by Dt. Kniegesellschaft	Web application	Diseases of the patellofemoral region	4-Oct-21		345.10 €	27
	Pain in extremities: Lower leg [fibula, tibia, and knee joint]					
	Luxation of the patella					
NichtraucherHelden-App	Apple App Store	Mental and behavioral disorders caused by tobacco: addiction syndrome	3-Jul-21		329.00€ (first prescription)	28
	Google Play Store				119.00€ (each renewal)	
ESYSTA App & Portal – Digitales Diabetesmanagement^b^	Apple App Store	Diabetes mellitus, type 1 and type 2	4-Jul-21	4-Oct-22	249.86 €	29
	Google Play Store					
	Web application					
Novego: Depressionen bewältigen	Web application	Depression	10-Oct-21		249.00 €	30
elevida	Web application	Multiple sclerosis	15-Dec-20		243.00 €	31
HelloBetter Stress und Burnout	Web application	Stress and burnout	11-Dec-21		235.00 €	32
HelloBetter Vaginismus Plus	Web application	Vaginism	11-Dec-21		235.00 €	
Kranus Edera	Apple App Store	Erectile dysfunction	18-Dec-21		235.00 €	
	Google Play Store					
Velibra	Web application	Agoraphobia; social phobias; panic disorder; generalized anxiety disorder	1-Oct-20		230.00 €	33
HelloBetter Panik	Web application	Agoraphobia	11-Dec-21		230.00 €	
		Panic disorder				
Selfapys Online-Kurs bei Generalisierter Angststörung	Apple App Store	Generalized anxiety disorder	19-Jun-21		228.50 €	34
	Google Play Store					
	Web application					
Somnio	Apple App Store	Nonorganic insomnia	22-Oct-20		224.99 €	35
	Google Play Store					
	Web application					
HelloBetter Diabetes und Depression	Web application	Diabetes mellitus, type 1 and type 2	11-Dec-21		222.99 €	36
Invirto (+ VR Goggles)	Apple App Store	Agoraphobia; social phobia; panic attacks	3-Dec-20		220.00 €	37
	Google Play Store					
M-sense Migräne^b^	Apple App Store	Migraine	16-Dec-20	4-Apr-22	219.99 €	38
	Google Play Store					
Novego^a^	Web application	Agoraphobia	24-Mar-23		219.98 €	39
		Social phobia				
		Special isolated phobia				
		Panic disorder				
Zanadio	Apple App Store	Obesity	22-Oct-20		218.00 €	40
	Google Play Store					
Selfapys Online-Kurs bei Depression	Apple App Store	Depression	16-Dec-20		217.18 €	41
	Google Play Store					
	Web application					
Deprexis	Web application	Depression	20-Feb-21		210.00 €	42
Vivira	Apple App Store	Arthrosis; joint pain and joint disease; osteochondrosis; other specified diseases of the spine and back; other biomechanical dysfunction	22-Oct-20		206.79 €	43
	Google Play Store					
Vorvida	Web application	Mental disorders caused by/due to alcohol	6-May-21		192.01 €	44
Kalmeda	Apple App Store	Tinnitus	25-Sep-20		189.00 €	45
	Google Play Store					
Mawendo	Web application	Diseases of the patella	9-Aug-21		119.00 €	46

*Notes:* DiGA listed in the descending order of price.

A more detailed description of each product and a summary of the clinical evidence of its positive care effects can be found in the DiGA Directory: https://diga.bfarm.de/de/verzeichnis. Sources: https://diga.bfarm.de/de; https://diga.bfarm.de/de/verzeichnis; https://www.diga-verzeichnis.de/digas. Last update: June 13, 2022.

a Indicates a provisional listing in the DiGA registry.

b Indicates that the app was later delisted.

While statutory health insurers must reimburse all DiGA in the BfArM’s Directory, only nine of the 33 DiGA listed through June 2022 were listed permanently at the time of launch. The other 24 were first listed provisionally and, according to the terms of such a listing, must provide additional evidence – via registered studies, as stated in the application – during their first year of reimbursement in order to move from provisional to permanent listing status (for more details on Germany’s Fast-Track, see the BfArM’s *Fast-Track Guide* (18)). As of October 2023 (when the data for this study were last updated), 26 of 49 DiGA were permanently listed, while four more had been delisted in the interim.

Secondly, and crucially for proponents of dynamic HTA, the pricing mechanism for DiGA induces an ongoing assessment of their value by obliging the contracting parties – DiGA manufacturers and Germany’s umbrella organization of statutory health insurers – to provide for “performance-based price components.” These components could be simple measurements, such as frequency of use, and/or may involve the development of DiGA-specific health indicators, such as sensor-derived measures or patient-reported outcomes.

## The use-case for “dynamic HTA” for SaMD

**Figure 1.**
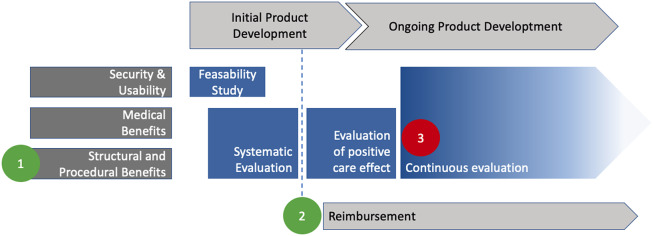
Ongoing evaluation of digital health applications in Germany.

Three core characteristics render the conduct of HTAs in the context of Germany’s Fast-Track pathway for DiGA unique:

1. the ability for manufacturers to choose from a range of research designs and outcome measures;
2. the nature of initial evidence generation, which can be delivered up to one year after a DiGA becomes reimbursable (facilitated by the “DiGA-Fast-Track” pathway described above);
3. incentives for continuous assessment of a DiGA’s benefit by obliging the parties that engage in price contracting to provide for performance-based price components of pricing/reimbursement. Such a continuous evaluation can be facilitated by using real-world data (RWD) and real-world evidence (RWE) (see Figure 1).

We explain these dynamic features for SaMD HTA and discuss the potential need to further develop a continuous assessment to allow for the ongoing improvement of DiGA via updates. Further, we discuss how RWD can be used to improve such evaluations.

### Breadth of positive-care effects

The breadth of “positive-care effects” that are legally acceptable for DiGA reimbursement is broader than what is normally considered in evaluations of traditional therapeutics and medical interventions, which are limited to clinical benefits, such as improvements in morbidity and/or mortality. To make digital products available that provide a wider range of benefits, the option to provide evidence of improvement in “structural and procedural effect” for patients has been introduced in Germany. Such improvements, if established, constitute sufficient criteria for the reimbursement of DiGA and include aspects such as health literacy, access to care, adherence, care coordination, and other patient-centered benefits that are not traditionally included in HTAs for other therapeutics (13).

Table 2 defines both clinical benefits and structural and procedural effects (Column I). Of 55 DiGA in our sample (including the six that became listed and then subsequently left the registry), 53 claimed medical benefits: 42 presented improvements in health status, four presented improvements in quality of life, and seven presented both. Nine of these apps provided further claims of improvement of structure and processes; however, only two became reimbursable on the basis of such a claim (2 and 6, respectively) alone (Column 2). The limited use of such patient-centered, clinical claims suggests that there is still ample potential to incorporate improvements in structure and processes, both in the design of DiGA as well as in their evaluations.

**Table 2.** Definitions and sample DiGA

Positive-Care Effects	DiGA (name of digital health application)
Medical Benefits	
1. Improvement of health status	Cara Care*, companion patella, deprexis, edupression*, elevida, elona therapy*, ESYSTA**, HelloBetter Diabetes und Depression, HelloBetter Panik, HelloBetter ratiopharm chronischer Schmerz*, HelloBetter Schlafen*, HelloBetter Stress und Burnout, HelloBetter Vaginismus Plus, Invirto, Kaia COPD*, Kaia Rückenschmerzen, Kalmeda, Kranus Edera, Mawendo, mebix*, Meine Tinnitus App*, Mindable, M-sense**, My7steps*, neolexon*, NeuroNation*, NichtraucherHelden-App, Novego: Ängste überwinden*, Novego: Depressionen bewältigen, Orthopy*, Oviva Direkt, PINK! Coach*, priovi*, re.flex*, Rehappy**, Selfapys Online-Kurs bei Binge-Eating-Störung, Selfapys Online-Kurs bei Bulimia Nervosa, Selfapys Online-Kurs bei chronischen Schmerzen*, Selfapys Online-Kurs bei Depression, Selfapys Online-Kurs bei Generalisierter Angststörung, Selfapys Online-Kurs bei Panikstörung**, sinCephalea*, Smoke Free – Rauchen aufhören*, somnio, velibra, Vitadio*, Vivira, vorvida, zanadio (49 minus 4)
2. Improvement of quality of life	Cara Care*, Endo-App*, Kaia COPD*, Levidex*, Mika**, M-sense**, Optimune*, Rehappy**, Selfapys Online-Kurs bei Generalisierter Angststörung, Selfapys Online-Kurs bei Panikstörung**, zanadio (11 minus 4)
3. Prolongation of survival	
4. Reduction in the duration of illness	
Improvement of structure and processes	
1. Coordination of treatment processes	
2. Alignment of treatment with guidelines and recognized standards	ProHerz*
3. Adherence	Rehappy**
4. Facilitation of access to care	
5. Patient safety	
6. Health literacy	CANKADO**, Cara Care*, edupression*, M-sense**, Rehappy** (5 minus 2)
7. Patient sovereignty/autonomy in health management	Kranus Edera, Mindable, M-sense**, Rehappy**, vorvida (5 minus 2)
8. Coping with disease-related difficulties in everyday life or	Cara Care*, Meine Tinnitus App*, Rehappy** (3 minus 1)
9. Reduction of therapy-related expenses and burdens for patients and their relatives	velibra

*Note:* * indicates a provisional listing in the DiGA registry. ** indicates that the app was later delisted. DiGA = Digitale Gesundheitsanwendung. For full definitions of each of the positive-care effects, see Section 4.1 of the DiGA Guide (15). Source: DiGA Registry https://diga.bfarm.de/de/verzeichnis.

Allowing for reimbursement of products that *only* provide structural and procedural benefits creates a market for manufacturers and products that provide the types of patient benefits that have not traditionally been considered in pharmaceutical or medical product evaluations and can help to ensure revenues for manufacturers while iterative software development and evidence generation proceed. The starting point of such a product development process may be SaMDs that cannot *yet* demonstrate medical benefits but can demonstrate other patient-relevant benefits based on early data. In particular, SaMD products with the potential to provide medical benefits, in the long run, can be made available to patients in the short run if structural improvements to care can be demonstrated during the initial evaluation period. For example, an application that increases health literacy could indirectly improve the quality of life (19) or add new features that directly provide medical benefits and then generate supporting evidence for these additional claims after becoming reimbursable.

### Nature of initial evidence generation

The nature of initial evidence generation is expected to differ for DiGA. While other medical products, such as pharmaceuticals, are typically approved based on completed clinical trials, clinical evidence for DiGA in the German regulatory setting can be delivered *up to one year after becoming reimbursable.* Further, the existing policies give DiGA manufacturers a large degree of flexibility in choosing research designs, explicitly accepting retrospective studies, such as cohort studies, as sufficient evidence if meaningful data are used and if the study population is comparable with the actual intended users.

This flexibility in the timing of delivery of clinical evidence allows DiGA manufacturers to scale up their evaluation efforts (in some cases, only) once the product is made widely available – facilitating market entry for small firms. In fact, most of the currently available DiGA have opted for small-scale trials for gaining reimbursement initially (20). Also, noteworthy is the comparator group selected in DiGA studies: By default, the fast-track process requires a manufacturer to compare the use of the DiGA against the “realities of care,” which also includes comparison to *the absence of care*, for example, in contexts during which patients usually wait for an extended period of time to begin a treatment or for orphan diseases for which no treatment is available.

Early access to reimbursement for qualifying SaMDs under the DiGA scheme is, however, not uncontroversial. In particular, the sickness funds (German statutory health insurers) that are required to reimburse for approved DiGA have voiced concerns around the introduction of the scheme (21). Two DiGAs that were initially added to the registry have already been removed from reimbursement after they failed to provide sufficient evidence for the benefit they claim to deliver (22). Whether early examples of DiGA that leave the market after the probation period is a sign that the system works – or an indicator of overspending on nonevidence-based apps (albeit only over a brief period of time) – remains an open question for the coming years.

### Continuous evaluation

While most medical devices and medicinal products are reimbursed at a fixed price typically negotiated between the manufacturer and health insurers, the legal framework regulating the DiGA system explicitly obligates the contracting partners to provide for performance-based price components of pricing/reimbursement. This means that even after successful demonstrations of benefits in the DiGA process, more evaluations are needed. Such evaluations require the selection of appropriate endpoints for ongoing measurement as well as a financial valuation of the savings (or costs) associated with an improvement (or deterioration) of these. This, in turn, lends itself to and incentivizes the development of scientific methods for evaluating both clinical outcomes and health economic measures using RWE, paving the way for (increasingly) value-based healthcare models (23).

A crucial feature of HTA in the context of SaMD products is that after the initial product launch and evaluation, there is both a need and an opportunity for ongoing product evaluation and modification. This dynamic evaluation is necessary, as products are expected to be updated (and improved) over time. Others have outlined the challenges and opportunities in software-driven devices, with dynamic product development highlighted among them (24). A SaMD product must be updated on an ongoing basis ranging from maintaining compatibility with operating systems or browser software to improving performance, fixing bugs, and releasing additional patient-benefiting modules.

The need for continuous evaluations of SaMDs and other medical interventions has been identified before (25; 26). Further, two recent policy reports on the new DiGA scheme recommend ongoing and “agile” evaluations to meet the requirements of continuously developed and refined DiGA (20; 23).

## Using RWE to facilitate dynamic HTA

The special features in Germany’s DiGA regulations (the breadth of positive-care effects, the unique and flexible nature of initial evidence generation, and the need for continuous evaluation) highlighted above will require new evaluation methods. One crucial factor for a successful widespread application of dynamic HTA will be the use of RWD and RWE.

FDA provides clear and helpful definitions of RWD and RWE, which we rely upon here:

“Real-world ***data*** are the data relating to patient health status and/or the delivery of health care routinely collected from a variety of sources.” (27) These may include data from electronic health records (EHRs), claims and billing activities from the healthcare delivery system, product and/or disease registries, patient-generated data (including from in-home use and digital devices such as wearable sensors), as well as data from other sources that are relevant to the health status, such as data or metadata from mobile devices (27).

Relatedly, but distinctly, “real-world ***evidence*** is the clinical evidence regarding the usage and potential benefits or risks of a medical product derived from analysis of RWD.” Generally speaking, “RWE can be generated by different study designs or analyses, including but not limited to, randomized trials, including large simple trials, pragmatic trials, and observational studies (prospective and/or retrospective)” (27)– that is, RWE explicitly covers many of the study design options that are outlined in the regulations that govern acceptable forms of clinical evidence for DiGA in the German market.

Indeed, RWD and RWE can support all three dynamic HTA features described above. First, SaMDs can be designed to record data to evaluate positive-care effects for users over time. For example, metadata from apps themselves can track the duration and frequency of use and therefore measure adherence to a prescribed treatment regimen that involves a SaMD product. Improvements in health status can also be documented via sensors and connected devices, such as smart glucometers for patients with type I diabetes. Other positive care effects can be identified from existing data sources. For example, health insurance claims data can be used to document reductions in illness duration and increases in patient survival. Finally, features such as in-app questionnaires lend themselves easily to the digital environment, providing a standardized framework for collecting data within the context of product use. In-app questionnaires can be used to document several relevant categories of positive-care effects ranging from established scales for measuring patient quality of life to nearly all measures of “structure and process” (see Table 2), such as facilitation of access to care, health literacy, patient sovereignty/autonomy in health management, and reducing therapy-related expenses and burdens for patients and their relatives. Of course, the scope for gathering RWD goes well beyond health insurance claims and in-app questionnaires, and in the future, the increasing digitization of health systems combined with the establishment and growth of patient registries will create new opportunities for the generation of RWD. Looking forward, RWD from a number of sources will be an important input to assessing the benefits and costs/savings associated with new healthcare technologies.

RWD and RWE employed as suggested above also have the potential to support initial evidence generation in the DiGA process. As noted, in order to gain reimbursement in Germany via the DiGA Fast-Track, some forms of initial evidence must be provided to the BfArM. RWE, for example, from claims data, can deliver this initial evidence at costs far below those associated with traditional RCTs. Most importantly, to make ongoing evaluations feasible and the corresponding HTAs truly dynamic, both RWD and RWE will be necessary because full RCTs are simply not feasible for every product update – nor would their requirement be desirable, as it would slow the pace of innovation and/or roll-out of improvements and additional beneficial features to patients. Indeed, many non-digital medical devices undergo incremental innovation, whereby new product versions can be released without a full clinical evaluation involving RCTs.

RWE has been widely used in many countries. Pongiglione et al. (28) provide a review of sources of RWD and to what extent they are known and used in medical, epidemiological, and economic research in 13 European countries. One example shows that cancer survival rates are higher for participants of an RCT versus an RWE cohort (29). The National Institute for Health and Care Excellence regularly accepts RWE in the evaluation of cancer drugs (30) as does FDA, which has published formal regulatory guidance on the use of RWD and RWE for studies of biologic drugs (31) and medical devices (32) as well as specific recommendations on the use of HER data in clinical investigations (33). Even though expert interviews from Germany reveal a generally positive attitude toward RWD and RWE (34), none of the DiGA approved to date have used this form of data and evidence generation, suggesting a clear opportunity for the introduction of new tools and data sources going forward. There are already important steps toward that goal with new tools currently developed for example in the framework of data fusion (35) while a new research data center for healthcare data currently established in Germany can serve as a hub for RWE studies.^1^

Of course, the use of RWE has many limits. Causal inference is more difficult than in RCTs and managing challenges such as patient selection, data representativeness, and data privacy/security will be key issues for the practical success of implementing RWE in the German context. Further, researchers have highlighted key areas that should be prioritized for the use of RWE in digital medical product evaluation and in the promotion of international harmonization of best evidentiary practices. These include the establishment of best practices around topics including missing data, study endpoints, comparator group(s), multimodal interventions, study question(s), equity, generalizability, confounders, and fit-for-purpose approaches (36). However, the promise of more and richer data, better tools, and more patient-centered data collection and product launches also provide an overwhelming promise for learning to implement RWE approaches thoughtfully. Institutionalizing continuous evaluation with RWE for SaMDs, however, could be the first step toward a broader movement of assessing benefits in healthcare systems both on the disease and system levels (37).

More broadly, pioneers of RWE for dynamic HTA in the SaMD setting may also garner insights from studying other approaches that take advantage of dynamic, ongoing data generation – and in particular, the use of RWD for health economic decision-making internationally. For example, “coverage with evidence development” (CED) has many parallels with the DiGA Fast-Track. Experts on CED have noted that when used “selectively” and for “innovative” interventions, this approach can “provide patient access…while data to minimize uncertainty are collected” (38). Other work on CED has described the types of settings in which such an approach is likely to be most appropriate, arguing for its use when “there are reasonable grounds for believing that a technology will offer significant benefits” and remaining uncertainty “around the clinical or cost effectiveness…can be overcome through evidence that can be generated in an appropriate time frame and is the main source of equivocality in a coverage decision” (39), a set of circumstances that also directly applies to the second core characteristic of HTAs in the context of Germany’s Fast-Track pathway for DiGA.

## Conclusion

Digital health applications and the accompanying demand for dynamic HTA present both a significant challenge and great opportunity for contemporary healthcare delivery. In Germany, new policies now allow for the use of both a broader set of research designs and more flexible approaches for their demonstration. However, early experience with evidence generation has shown that manufacturers are still hesitant to focus on nontraditional endpoints and nontraditional evidence-generation strategies. In particular, recent changes to German policy have facilitated reimbursement of SaMD products that do not necessarily fit with either current HTA approaches nor are they well-matched to the unique characteristics and additional needs of digital health applications (in particular, ongoing development and, as a corollary, a need for ongoing evaluation).

As such, a new, *dynamic HTA* will be important both to facilitate continuous improvement and ongoing reimbursement of innovative healthcare solutions as well as the basis for their fair, evidence-based, and efficient reimbursement after launch. Additionally, the approaches presented here may have implications for the development of HTA for non-digital products such as orphan drugs, where approval decisions may be made based on limited evidence and subsequently supported by RWD and RWE from routine medical practice (e.g., claims data) or registries.

The next generation of HTAs for SaMD in general – and for DiGAs in Germany in particular – will need to take advantage of new sources of RWD and methodological innovations, including improvements and best practices in the use of RWD, while managing the challenges unique to using RWE. If successful, such approaches will facilitate patient access to demonstrably beneficial SaMD products and ensure that their prices are value-based and, by doing so, improve the healthcare delivery system for all parties.

## Footnotes

1
https://www.forschungsdatenzentrum-gesundheit.de/das-fdz.
